# An In-Silico Evaluation of Anthraquinones as Potential Inhibitors of DNA Gyrase B of *Mycobacterium tuberculosis*

**DOI:** 10.3390/microorganisms10122434

**Published:** 2022-12-08

**Authors:** Juliana Carolina Amorim, Andrea E. Cabrera Bermeo, Viviana E. Vásquez Urgilés, Maritza R. Martínez León, Juan M. Carpio Arévalo

**Affiliations:** Academic Unit of Health and Wellness, Catholic University of Cuenca, Cuenca 010105, Ecuador

**Keywords:** *Mycobacterium tuberculosis*, DNA gyrase B, anthraquinones, aminotriazole moiety, pharmacokinetic profile, molecular dynamics

## Abstract

The World Health Organization reported that tuberculosis remains on the list of the top ten threats to public health worldwide. Among the main causes is the limited effectiveness of treatments due to the emergence of resistant strains of *Mycobacterium tuberculosis*. One of the main drug targets studied to combat *M. tuberculosis* is DNA gyrase, the only enzyme responsible for regulating DNA topology in this specie and considered essential in all bacteria. In this context, the present work tested the ability of 2824 anthraquinones retrieved from the PubChem database to act as competitive inhibitors through interaction with the ATP-binding pocket of DNA gyrase B of *M. tuberculosis*. Virtual screening results based on molecular docking identified 7122772 (*N*-(2-hydroxyethyl)-9,10-dioxoanthracene-2-sulfonamide) as the best-scored ligand. From this anthraquinone, a new derivative was designed harbouring an aminotriazole moiety, which exhibited higher binding energy calculated by molecular docking scoring and free energy calculation from molecular dynamics simulations. In addition, in these last analyses, this ligand showed to be stable in complex with the enzyme and further predictions indicated a low probability of cytotoxic and off-target effects, as well as an acceptable pharmacokinetic profile. Taken together, the presented results show a new synthetically accessible anthraquinone with promising potential to inhibit the GyrB of *M. tuberculosis*.

## 1. Introduction

Tuberculosis is a respiratory disease caused by *Mycobacterium tuberculosis* [1]. The disease currently affects about 10 million people worldwide, with an average of 1.3 million deaths per year [2]. Even with the introduction of the BCG vaccine more than a century ago, the global rate of infection with *M. tuberculosis* is one in three people [3]. In addition, the bacteria can escape from the cells of the immune system and remain dormant in old lesions which results in ineffective and prolonged treatment [3,4]. The treatment currently standardized by the World Health Organization as the first line against drug-susceptible *M. tuberculosis* infection is for six months and is effective in approximately 85% of cases [2,4]. It initially consists of the administration of ethambutol, isoniazid, pyrazinamide and rifampicin for two months, followed by four months using isoniazid and rifampicin [3,4]. Among the main causes that prevent a successful treatment are the incorrect prescription of drugs or the lack of them in the health units, and also the low adherence to treatment by patients [2]. As a consequence, multidrug-resistant strains of *M. tuberculosis* emerge and adapt, becoming more efficient in the infectious process and the evolution of tuberculosis [5].

Among the targets considered essential for bacterial survival and validated for antituberculosis drug discovery is DNA gyrase [6]. This enzyme is representative of the type II topoisomerases in bacteria and is responsible for catalyzing the introduction of negative DNA supercoils in an ATP-dependent manner [7]. The main activities of this enzyme are related to replication, transcription, and also recombination [8]. However, in *M. tuberculosis*, besides these functions, this enzyme is uniquely responsible for decatenation, which in other bacteria is carried out by DNA topoisomerase IV [9]. DNA gyrase is composed of two subunits called GyrA and GyrB, which form an A2B2 heterotetramer when active. While GyrA is responsible for breaking and reunion of the DNA strands, GyrB promotes the hydrolysis of ATP to provide energy for DNA supercoiling [7,8]. Among the main inhibitors described for GyrA are quinolones (e.g., ciprofloxacin), antibiotics responsible for interfering with the bacterial DNA-gyrase-DNA complex [10]. On the other hand, some of the main inhibitors described for GyrB, such as aminocoumarins [11], have an affinity for the ATP-binding pocket, acting competitively with this molecule and thus preventing its hydrolysis [12]. In addition, a wide variety of promising molecules have been reported in silico, in vitro and in vivo assays, such as the aminopyrazinamides [13], bithiazoles [14], indazoles [15], pyrrolamides [16], and pyrazoltiazoles [17].

Even though many described inhibitors for GyrB are being frequently tested, a considerable number of these have not been successful in clinical trials [12]. From this limitation, it becomes necessary to expand the search for new inhibitors for this target [18]. Compounds that show promising potential for a large number of therapeutic applications are phytochemicals [19,20,21,22,23,24], which are structurally diverse and complex groups of molecules [25]. Among the phytochemicals, anthraquinones are considered promising scaffolds for the development of potential inhibitors of nucleotide-binding proteins [26]. In addition, there are examples of anthraquinones that are approved drugs such as mitoxantrone used in chemotherapy and multiple sclerosis [27] or the food additive carmine, which also has the anthraquinone scaffold in its structure [28]. In fact, several anthraquinones have shown activity against Gram-positive and Gram-negative bacteria [29,30,31] and even against *M. tuberculosis,* as in the case of 3-hydroxy-1-methoxyanthraquinone-2-aldehyde [32]. In this context, the present work evaluated through in silico approaches the affinity of 2824 anthraquinones for the ATP-binding pocket of *M. tuberculosis* GyrB (*Mt*GyrB) to identify the most suitable chemical substituents to develop potential inhibitors based on the anthraquinone backbone.

## 2. Methods

### 2.1. Target and Ligands Preparation 

For the present work the *Mt*GyrB crystal RCSB PDB 3ZKB (resolution: 2.9 Å, free R-value: 0.240, working R-value: 0.182, observed R-value: 0.184 [33]) was used with the 216-239 loop region computationally rebuilt as described in a previous publication [34] and hereinafter referred to as 3ZKBL (3ZKB loop rebuilt). This target was prepared using the Dock Prep module of the UCSF Chimera-1.16 [35], then processed by the SPORES 1.3 tool using default parameters and saved in MOL2 format. 

The chemical structures of the 2824 anthraquinones were obtained from the PubChem database and downloaded in SDF format in March 2022. This database was generated from the substructure of the anthraquinone backbone and applied the following filters: rotatable bonds: maximum 10, hydrogen bond donors: maximum 5, hydrogen bond acceptors: maximum 10 and in the data source category: chemical vendors, to prioritize readily accessible molecules. Finally, the database was filtered to include structures with a molecular weight below 350 g/mol to restrict the search to aglycones and exclude dimeric structures and heterosides of anthraquinones. The ligand co-crystallized with 3ZKB, ANP, (33113([[[[(2R,3S,4R,5R)-5-(6-aminopurin-9-yl)-3,4-dihydroxyoxolan-2-yl]methoxy-hydroxyphosphoryl]oxy-hydroxyphosphoryl]amino]phosphonic acid) was used as control. Subsequently, to prepare the ligands for virtual screening, hydrogen atoms were assigned to the structures at pH 7.4 and generated their respective 3D structures. The minimization of these ligands was then performed with the MMFF94 force field [36] using the steepest descent geometry optimization with 500 steps, followed by a conjugate gradient algorithm with default parameters and transformed into MOL2 format, all using Open Babel-3.1.1 software [37].

### 2.2. Molecular Docking-Based Virtual Screening Analyses

The molecular docking-based virtual screening analyses were performed using Protein-Ligand ANT System-1.2 software (PLANTS-1.2) [38]. A 20 Å radius centering the coordinates of the ATP-binding pocket of 3ZKBL was set to x = −26.86, y = −27.11, and z = 17.77. The search speed was set to one and the scoring function was selected as ChemPLP. The clustering RMSD was set to 2.0 Å and all docking scores were calculated by the default scoring function, the other parameters were kept according to the software recommendations.

### 2.3. Pharmacokinetic, Cytotoxicity and Synthetic Accessibility Predictions

To obtain information on the pharmacokinetic properties of the most promising molecules and also of the reference compounds, predictions were made using the Swiss-ADME server [39], (http://www.swissadme.ch/ (accessed on 1 March 2022)). The properties evaluated were gastrointestinal absorption, permeability across the blood-brain barrier, potential transport by P-gp (P-glycoprotein) and the likelihood of inhibition of cytochromes related to drug metabolism. 

The cytotoxicity of the most promising compounds and also of the reference anthraquinone were evaluated using the ProTox-II server [40], (https://tox-new.charite.de/protox_II/index.php?site=compound_input (accessed on 14 November 2022).

Prediction of the synthetic accessibility of the new designed compound was performed using the Ambit-SA software [41]. The algorithm of the software uses four different descriptors to represent different structural and topological features: molecular complexity, steric chemical complexity, and the complexity due to the presence of fused and bridged systems. The results are presented as scores ranging from 0 to 100, where the value 100 represents the most easily synthesizable molecule.

### 2.4. Target Fishing Predictions

Target fishing analyses to comparatively evaluate the ability of the most promising molecule and the reference compounds to interact with human targets were performed using the Swiss Target Prediction server [42] (http://www.swisstargetprediction.ch/ (accessed on 1 March 2022)). For these analyses, the SMILES codes of the anthraquinones were generated and used as input files. The results are presented as scores ranging from 0 to 1, where the value 1 corresponds to the most likely target of the query molecule.

### 2.5. Molecular Dynamic Simulations

Molecular dynamics simulations (MDS) were performed using the GROMACS-2021.1 software [43] to better understand the interactions and stability of 3ZKBL and their complexes with selected ligands. All simulations were performed using the CHARMM 36 all-atom force field [44]. The transferable water model of intermolecular potential 3P was used for solvation by selecting a periodically corrected cubic box using a minimum distance of 1 nm. Later, the system was neutralized by adding Na^+^ and Cl^−^ ions, to eliminate the initial steric shocks, 100,000 energy minimization steps were conducted using the steepest descent algorithm. The system was equilibrated during 500 and 5000 ps at 310 K and 1 bar pressure in the NVT and NPT arrays, respectively. The runs were conducted during 100 ns and the coordinates were saved every 10 ps. The Particle Mesh Ewald algorithm [45] was used to analyze the long-range electrostatic interactions and the LINCS algorithm [46] was used to regulate the covalent bonds. The Leap-frog algorithm and Berendsen coupling [47] were used to control the pressure and temperature.

### 2.6. Binding Free Energy Calculation

To complement the MDS analyses, binding free energy calculations were performed using a single trajectory based on the MMPBSA method [48], using the gmx-MMPBSA 1.5.2 software [49]. To conduct the calculations, the results of the last 50 ns of the MDS performed with 3ZKBL and the selected anthraquinones were extracted, which were the equivalent of 2500 snapshots for each simulation. The parameters that were used to calculate the binding free energies were inp = 1, istrng = 0.15, and indi = 2, the other parameters were kept according to the software recommendations.

### 2.7. Visualizations of Molecular Docking and Molecular Dynamics Results 

The structures of anthraquinones were designed using the Marvin JS web server. 2D (https://marvinjs-demo.chemaxon.com/latest/ (accessed on 1 March 2022)) interaction diagrams of protein-ligand complexes were generated with the PoseView web server [50] (http://poseview.zbh.uni-hamburg.de (accessed on 1 March 2022)). The 3D diagrams were visualized using the software Discovery Studio Visualizer-2021. MDS analyses were visualized with GROMACS scripts in conjunction with Python scripts using the NumPy, Pandas, Matplotlib, Seaborn and Pytraj libraries. The RMSD, RMSF and Rg representations were generated from the alpha-carbon of the protein in the presence or absence of the ligands, while the hydrogen bonds were generated from the protein-ligand complexes.

## 3. Results and Discussion

### 3.1. Virtual Screening of Anthraquinones

The search for *Mt*GyrB inhibitors has been a collaborative effort between academia and industry for many decades [12] isolating and synthesizing a wide variety of inhibitors [51,52,53]. In parallel, there have also been considerable advances in the availability of crystallographic structures of *Mt*GyrB [33], which have allowed advances in silico studies to the discovery of potential inhibitors [54,55,56].

In the present work, 2824 anthraquinones, identified by their PubChem ID, were ranked based on their best scores (BS) from molecular docking-based virtual screening (Supplementary Table S1). Among the ten best ranked anthraquinones are BS-1 (7122772, *N*-(2-hydroxyethyl)-9,10-dioxoanthracene-2-sulfonamide), BS-2 (85992490, (9, 10-dioxoanthracen-2-yl) methanesulfonate), BS-3 (3794639, 1-[(5-nitropyridin-2-yl)amino]anthracene-9,10-dione), BS-4 (133109345, (1,4-dihydroxy-9,10-dioxoanthracen-2-yl) hydrogen sulfate), BS-5 (94940647, 1-(9, 10-dioxoanthracen-2-yl)triazole-4-carboxylic acid), BS-6 (108793505, *N*-(9,10-dioxoanthracen-1-yl)-6-oxo-1H-pyridazine-3-carboxamide), BS-7 (7122774, *N*,*N*-dimethyl-9,10-dioxoanthracen-2-sulfonamide), BS-8 (625367, 6-chloro-9,10-dioxoanthracene-2-carboxylic acid), BS-9 (100282, 1,4-bis(2-aminoethylamino)anthracene-9,10-dione) and, BS-10 (123132836, (E)-3-[5-(9,10-dioxoanthracen-1-yl)furan-2-yl]prop-2-enoic acid). The results in Table 1 show that, of these anthraquinones, six derivatives have substituents attached to carbon 2, of which four are sulfur-oxygen containing groups, such as sulfonamide (BS-1 and BS-7), sulfonate (BS-2) and sulfate (BS-4). Another three anthraquinones have substituents at position 1 (BS-3, BS-6 and BS-10) and one have groups in position 1 as well as in position 4 (BS-9).

**Figure 1:**
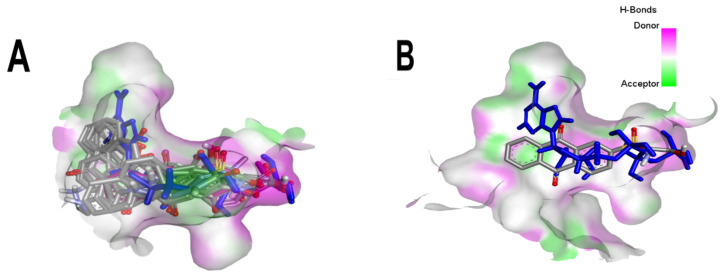
Superposition of the best poses of the best-scored anthraquinones compared to the crystallographic pose of ANP in the catalytic site of *Mt*GyrB. (**A**) Superposition of the ten best poses of the best-scored anthraquinones compared to the crystallographic pose of ANP. (**B**) Superposition of the best-scored one (BS-1) anthraquinone compared to the crystallographic pose of ANP. BS-1 (7122772), BS-2 (85992490), BS-3 (3794639), BS-4 (133109345), BS-5 (94940647), BS-6 (108793505), BS-7 (7122774), BS-8 (625367), BS-9 (100282) and BS-10 (123132836). ANP (33113) is highlighted in dark blue. 3D hydrogen bond surface representation was performed with Discovery Studio Visualizer.

It is important to note that all the above-mentioned substituents have hydrogen donor and acceptor groups. In addition, although the binding affinity of anthraquinones is slightly lower than that of ANP (−112.5), all their polar substituents and phosphate groups, respectively, are oriented toward the interior of the catalytic site in an analogous manner (Table 1 and Figure 1A). Considering the higher binding affinity and frequency of sulfur-oxygen-containing groups present at carbon 2 of the aforementioned anthraquinones, the sulfonamide derivative, BS-1, was selected for further analysis. 

### 3.2. Comparative Analyses of the ANP and BS-1 Poses

**Figure 2:**
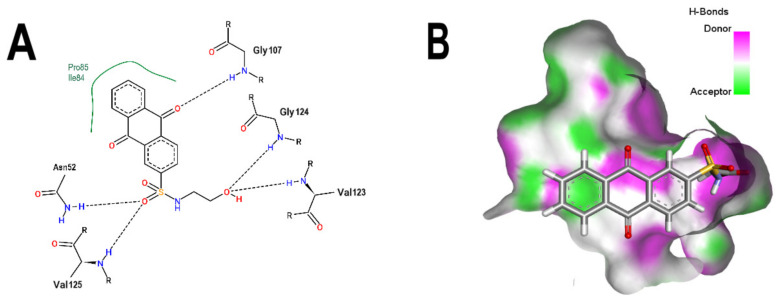
Analysis of the best pose of BS-1. (**A**) 2D interaction diagram of the 3ZKBL-BS-1 complex. (**B**) 3D representation of hydrogen bond surface of the 3ZKBL-BS-1 complex. 2D interaction diagram was performed with PoseView and 3D hydrogen bond surface representation was performed with Discovery Studio Visualizer.

**Figure 3:**
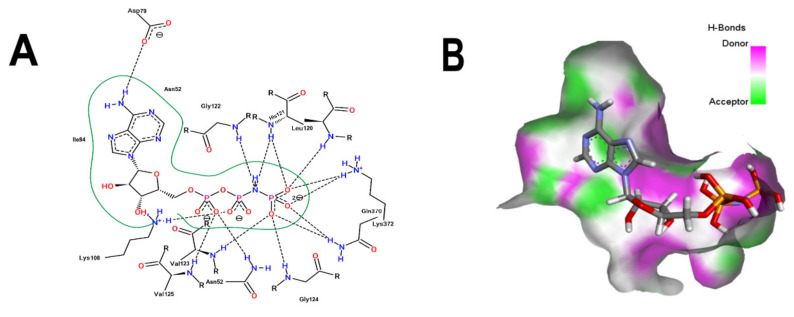
Analysis of the crystallographic pose of ANP. (**A**) 2D interaction diagram of 3ZKBL-ANP complex. (**B**) 3D representation of hydrogen bond surface of 3ZKBL-ANP complex. 2D interaction diagram was performed with PoseView and 3D hydrogen bond surface representation was performed with Discovery Studio Visualizer.

Figure 1B shows that the sulfonamide linked to the hydroxyethyl substituent of BS-1 projects deeply into the ATP-binding pocket of 3ZKBL like that of the phosphate groups of the ANP. As shown in Figure 2A,B, BS-1 formed hydrogen bonds with the amino acids Asn52, Gly107, Val123, Gly124 and Val125 of 3ZKBL. Except for Gly107, all these hydrogen bonds are also established among the ANP and 3ZKBL (Figure 3A,B). However, while ANP forms a hydrogen bond between the amino group of its adenine and the carboxylate group of Asp79, which is a key residue to the bacterial GyrB function [57,58], BS-1 shows no interaction with this amino acid, Figure 2A.

**Figure 4:**
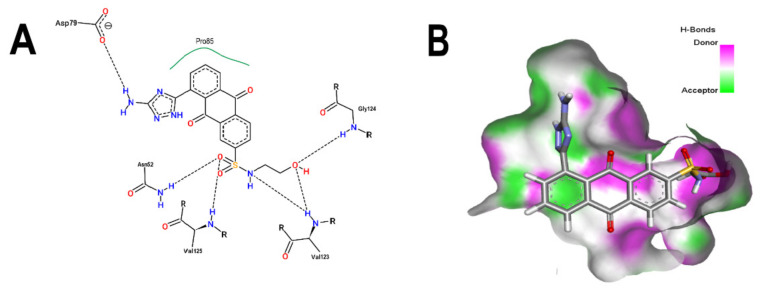
Analysis of the best pose of ATD (aminotriazole anthraquinone derivative). (**A**) 2D interaction diagram of the 3ZKBL-ATD complex. (**B**) 3D representation of hydrogen bond surface of 3ZKBL in complex with ATD. 2D interaction diagram was performed with PoseView. 3D hydrogen bond surface representation was performed with Discovery Studio Visualizer.

Based on the above analyses, the next step was to introduce modifications to the structure of BS-1 in an attempt to optimize its interactions with the enzyme, including the formation of a hydrogen bond with Asp79. For the design of this new derivative, priority was given to the selection of a moiety that, when fused to carbon 8 of the anthraquinone backbone of BS-1, would be able to provide hydrogen donor and acceptor groups in a manner analogous to adenine in nucleotides such as ANP. To this end, an aminotriazole was incorporated, which is a moiety present in certain drugs [59], and some promising antitubercular agents [60]. In fact, this moiety has been reported previously as a substituent in the anthraquinone core (PubChem ID: 153843066), but there is no information on the potential biological activities of this derivative. The result of the PLANTS analysis shown in Figure 4A confirms that the amino group of the triazole ring of this new derivative (hereafter referred to as ATD, aminotriazole anthraquinone derivative) can form a hydrogen bond with Asp79, Figure 4B. In addition, the inclusion of this moiety causes only a small change in the RMSD values of the anthraquinone backbone compared to BS-1 (0.342 Å). Furthermore, this aminotriazole derivative also exhibits an increased binding affinity to reach a value of −107.5 in the PLANTS score. To evaluate the synthetic accessibility of ATD, the Ambit-SA software was used, this shows a score of 60.41 indicating that ATD is a synthesizable anthraquinone.

### 3.3. Pharmacokinetic and Cytotoxicity Analyses 

**Figure 5:**
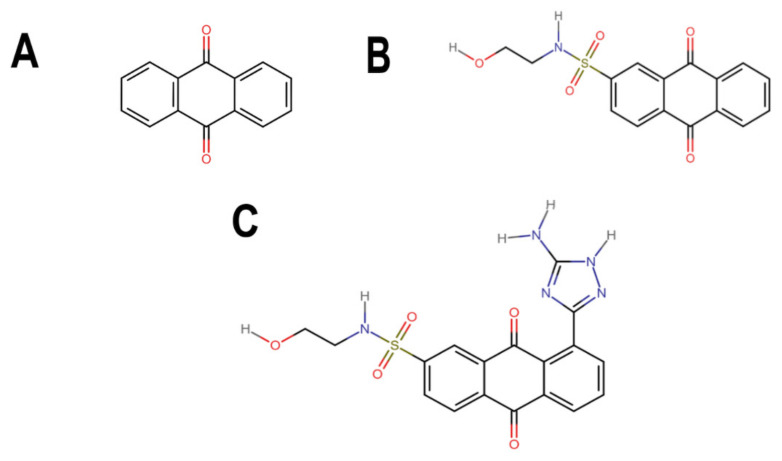
Chemical structures of molecules used in prediction analysis. (**A**) The anthraquinone core without functional groups, AC. (**B**) The best-scored anthraquinone, BS-1 and (**C**) The designed anthraquinone, ATD.

To obtain information on some of the pharmacokinetic properties of ATD, analyses were performed using the Swiss ADME server. Additionally, for comparative purposes, these same predictions were performed for the anthraquinone core without functional groups (hereinafter called AC, Figure 5A) and for the parent molecule, BS-1 (Figure 5B). The predictions reveal that ATD (Figure 5C) is a P-gp substrate and has lower gastrointestinal absorption than AC and BS-1, thus its parenteral administration may be necessary for further in vivo experiments. In addition, unlike AC, ATD does not have properties to cross the blood-brain barrier (BBB), which could reduce the risk of adverse effects at the central nervous system level [61].

Importantly, while AC could inhibit some cytochromes, which has even been demonstrated experimentally [62], ATD would not be a cytochrome inhibitor (Table 2). This last result suggests that ATD would not have interactions caused by inhibition of these cytochromes that metabolize a wide variety of drugs [63]. Overall, these analyses show that ATD exhibits a pharmacokinetic profile suitable for further in vivo experiments.

To complement these analyses, a prediction of the cytotoxicity of these molecules was performed, as shown in Table 3. The results show that all three molecules, AC, BS-1 and ATD, present probabilities higher than 60% of being inactive as cytotoxic agents, which supports that ATD could be considered a promising molecule for future experiments.

### 3.4. Target Fishing Analysis

Given that the off-target effects of drugs can provoke adverse effects [64] and knowing that several anthraquinones interact with human enzymes [65], target fishing analyses were performed to identify possible human targets for ATD, AC and BS-1 (Supplementary Table S2). The results in Table 4 show that, compared to the high probability (0.898) of AC interacting with the phosphatase Cdc25B, which is involved in the activation of cyclin-dependent kinases at key stages of the cell cycle [66], neither BS-1 nor ATD would affect this activity. In fact, these results show that ATD would not have human protein targets, which is relevant considering that ATD is proposed as an antibacterial candidate and therefore it should not affect human protein functions.

### 3.5. Molecular Dynamic Simulations Analyses

**Figure 6:**
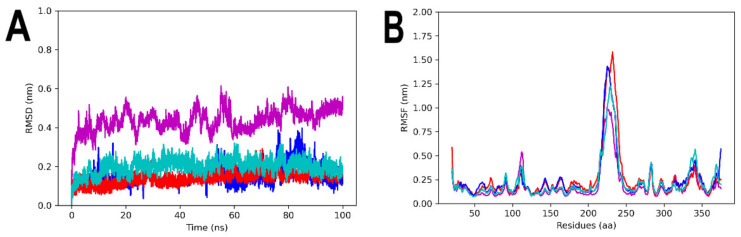
MDS analyses of apo-3ZKBL and 3ZKBL complexed with ANP, BS1, and ATD. (**A**) RMSD values. (**B**) RMSF values. The colors magenta, red, dark blue and light blue represent apo-3ZKBL, 3ZKBL-ANP, 3ZKBL-BS-1 and 3ZKBL-ATD respectively. The analyses were performed with GROMACS.

The MDS was performed to evaluate the stability and interactions of the complexes formed between 3ZKBL and the selected ligands. The first analysis to evaluate the stability of apo-3ZKBL consisted of calculating its RMSD, which revealed a mean value of 0.3 nm, with fluctuations between 0.3 and 0.5 nm throughout the simulation. Importantly, the 3ZKBL-BS-1, 3ZKBL-ANP and 3ZKBL-ATD complexes show lower RMSD values compared to the apo form. All complexes exhibited an RMSD less than 0.2 nm (Figure 6A), which reveals that these ligands form stable complexes with the enzyme.

To identify the amino acids with higher fluctuations of apo-3ZKBL and their complexes, their RMSF values were calculated. The results show more flexibility at residues 216–239 (a region that corresponds to the rebuilt loop). The maximum values of RMSF corresponding to these residues were 1.0, 1.3, 1.4, and 1.6 nm, respectively for apo-3ZKBL, 3ZKBL-ATD, 3ZKBL-BS-1 and 3ZKBL-ANP. However, the loop containing the residues responsible for interacting with the ATP (amino acids 103–122), showed greater flexibility in apo-3ZKBL (RMSF of 0.5 nm) compared to 3ZKBL-ANP (RMSF of 0.4 nm) and to 3ZKBL-BS-1 and 3ZKBL-ATD which showed almost the same value (RMSF of 0.35 nm), Figure 6B. These results suggest that the ligands stabilize the enzyme, and also that the greatest fluctuations are in regions corresponding to loops, where high flexibility in GyrB is already expected [67].

**Figure 7:**
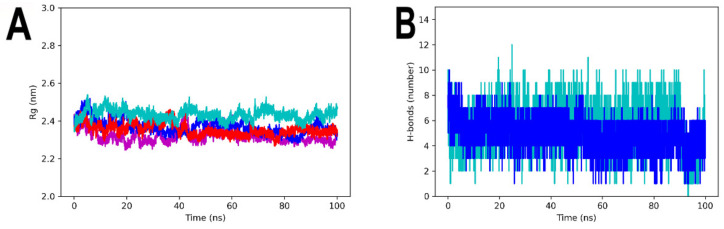
MDS analyses of apo-3ZKBL and 3ZKBL complexed to ANP, BS1, and ATD (**A**) Rg values. (**B**) Hydrogen bond number. The colors magenta, red, dark blue and light blue represent apo-3ZKBL, 3ZKBL-ANP, 3ZKBL-BS-1 and 3ZKBL-ATD, respectively. The analyses were performed with GROMACS.

Analyzing the Rg values of each of the systems over the simulation time, it can be seen that the complexes, as well as the apo-3ZKBL form, have very similar compactness values with average Rg values of 2.4 nm throughout the run. The maximum Rg value of apo-3ZKBL and 3ZKBL-ATD was 2.55 and 2.57 nm, respectively from the initial 5 ns of the simulation. For the 3ZKBL-ANP and 3ZKBL-BS-1 complexes, the maximum Rg values were 2.47 and 2.45 nm, respectively near the 10 ns of the simulation (Figure 7A).

Regarding the hydrogen bonds formed between the enzyme and the ligands, it can be observed that 3ZKBL and BS-1 establish a maximum of 10 hydrogen bonds at the beginning of the run, while an average of six to eight interactions occurred intermittently during the remaining run time. While the complex formed between 3ZKBL and ATD establishes up to 12 hydrogen bonds in 25 ns, followed by nine and 10 bonds intermittently maintained in the remaining analysis time (Figure 7B).

**Figure 8:**
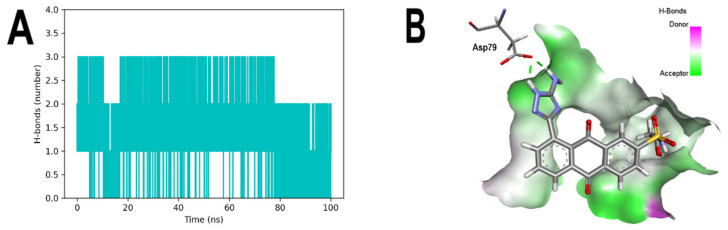
MDS analyses of 3ZKBL complexed to ATD. (**A**) Hydrogen bonds between Asp79 and ATD in the 3ZKBL-ATD complex. (**B**) Snapshot at 100 ns of 3ZKBL-ATD complex highlighting the hydrogen bonds of ATD with Asp79. The hydrogen bond analysis was performed with GROMACS. 3D hydrogen bond surface representation was performed with Discovery Studio Visualizer.

In addition, the formation of up to two and three hydrogen bonds between the Asp79 of 3ZKBL and the aminotriazole moiety of the ATD is also confirmed during most of the run (Figure 8A), which can also be observed in the snapshot obtained in the 100 ns run (Figure 8B).

### 3.6. Binding Free Energy Calculation

The result of binding free energy calculations presented in Table 5 shows an increase of ΔG (Total) of the 3ZKBL-ATD (−34.96 kcal/mol) compared to that of the 3ZKBL-BS-1 complex (−25.36 kcal/mol), which corroborates that the aminotriazole moiety enhances the affinity of the ligand for the enzyme. The decomposition of the binding energy shows an increment due to Van der Waals interactions, ΔE (Vdw), with values of −35.18 and −45.50 kcal/mol for the complex with BS-1 and ATD, respectively. The electrostatic interactions in the complex with BS-1 present unfavorable contributions with ΔE (Ele) of 73.27 kcal/mol, while ATD showed values of −30.83 kcal/mol. It was also observed that the polar solvation energy shows favorable values in BS-1, ΔE (Polar, Solv) of −59.40 kcal/mol and unfavorable ones in ATD with a value of 46.02 kcal/mol, it therefore presented opposite results to the results of the electrostatic interactions. 

## 4. Conclusions

As *M. tuberculosis* remains a threat to human health, mainly due to the limited effectiveness of existing treatments to circumvent multidrug-resistant events, the search for new molecules that can act as potential antibiotics are needed. In this work, the ability of 2824 anthraquinones to inhibit *Mt*GyrB through association with the ATP-binding pocket was analyzed. The molecule 7122772 (*N*-(2-hydroxyethyl)-9,10-dioxoanthracene-2-sulfonamide, BS-1) exhibited the best binding energy of *Mt*GyrB of the entire dataset. However, the aminotriazole anthraquinone derivative (ATD), which interacts with the key amino acid Asp79 of *Mt*GyrB, outperformed BS-1 in binding energy, pharmacokinetic profile and also exhibits a lesser probability of off-target effects. In addition, in MDS analyses, ATD complexed with *Mt*GyrB shows high stability and the binding free energy calculations exhibit better inhibitory potential than BS-1. Collectively, the results obtained in the present work reveal ATD as a new potential *Mt*GyrB inhibitor based on the anthraquinone backbone which deserves further experimental validation.

## Figures and Tables

**Table 1:** Structure and binding affinity of the best-scored anthraquinones and co-crystal ANP. BS-1 (7122772), BS-2 (85992490), BS-3 (3794639), BS-4 (133109345), BS-5 (94940647), BS-6 (108793505), BS-7 (7122774), BS-8 (625367), BS-9 (100282), BS-10 (123132836), and ANP (33113). These analyses were performed with PLANTS-1.2 software. Scores in PLANTS are dimensionless.

Score	Structure	Binding Affinity
BS-1	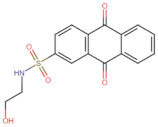	−105.4
BS-2	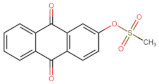	−103.6
BS-3	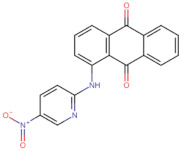	−103.2
BS-4	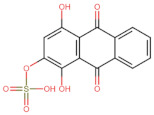	−102.4
BS-5	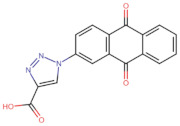	−102.4
BS-6	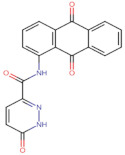	−102.4
BS-7	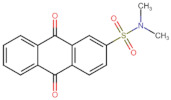	−102.4
BS-8	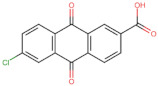	−101.4
BS-9	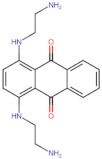	−101.4
BS-10	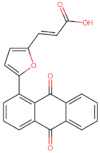	−101.0
ANP	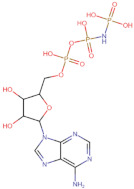	−112.5

**Table 2:** Pharmacokinetic analyses of AC, BS-1 and ATD. These analyses were performed with the Swiss ADME server.

Pharmacokinetic Analyses			
	AC	BS-1	ATD
GI absorption	High	High	Low
BBB permeant	Yes	No	No
P-gp substrate	No	No	Yes
CYP1A2 inhibitor	Yes	No	No
CYP2C19 inhibitor	Yes	No	No
CYP2C9 inhibitor	No	No	No
CYP2D6 inhibitor	No	No	No
CYP3A4 inhibitor	No	No	No

**Table 3:** Cytotoxicity predictions for AC, BS-1 and ATD. Probability values range from 0 to 1. Cytotoxicity analyses were performed with the ProTox-II server.

Cytotoxicity Prediction	Probability
AC	Inactive (0.85)
BS-1	Inactive (0.72)
ATD	Inactive (0.66)

**Table 4:** Target fishing analyses for AC, BS-1 and ATD. The results show the top five targets of AC, BS-1 and ATD ranked according to their probability of being targets of each molecule. Probability values range from 0 to 1. Target fishing analyses were performed with Swiss Target Prediction.

Target Fishing Analyses	Probability
Targets of AC	
Dual specificity phosphatase Cdc25B	0.89
Acyl coenzyme A: cholesterol Acyltransferase 1	0.06
Apoptosis regulator Bcl-2	0.06
Casein kinase I alpha	0.06
Caspase-3	0.06
Targets of BS-1	
Thymidine kinase cytoplasmatic	0.10
Thymidine kinase mitochondrial	0.10
Alkaline phosphatase	0.10
P-glycoprotein 1	0.10
Tyrosine-protein kinase ABL	0.10
Targets of ATD	
3-phosphoinositide-dependent protein kinase-1	0.00
78 kDa glucose-regulated protein	0.00
Adenosine A1 receptor	0.00
Adenosine A2a receptor	0.00
Adenosine A3 receptor	0.00

**Table 5:** Binding free energy calculations extracted from the single trajectory of MDS analyses of the 3ZKBL-BS-1 and 3ZKBL-ATD complexes. The analyses were performed with gmx-MMPBSA-1.5.4 software.

Energy Decomposition (Kcal/mol) ± Standard Deviation		
	BS-1	ATD
ΔE (Vdw)	−35.18 ± 1.95	−45.50 ± 2.57
ΔE (Ele)	73.27 ± 6.41	−30.83 ± 3.34
ΔE (Polar, Solv)	−59.40 ± 4.50	46.02 ± 2.94
ΔE (Non-Polar, Solv)	−4.03 ± 0.12	−4.64 ± 0.11
ΔG (Total)	−25.36 ± 3.11	−34.96 ± 3.49

## Data Availability

Not applicable.

## Supplementary Materials

The following supporting information can be downloaded at: https://www.mdpi.com/article/10.3390/microorganisms10122434/s1, Table S1: Molecular docking-based virtual screening of 2424 anthraquinones on the nucleotide-binding pocket of *Mt*GyrB. Table S2: AC, BS-1 and ATD target prediction.
